# Supplementary material for: Temporal Structure in Sensorimotor Variability: A Stable Trait, But What For?
Source: Comput Brain Behav. 2023 Jan 3:1–38. Online ahead of print. doi: 10.1007/s42113-022-00162-1 (PMC9810256; doi:10.1007/s42113-022-00162-1)
Supplement: Supplementary file 1 — Supplementary file1 (DOCX 61 KB) [file 42113_2022_162_MOESM1_ESM.docx]

**Supplementary Materials A**

*Table A1. Full list of the correlation coefficients (r-value) with corresponding 95% CI ([lower, upper]) and Bayes Factor (BF10) for the within-task repeatability of cognitive tasks, as displayed in Figure 6.*

| **Measure** | **Task** | **r-value** | **lower** | **upper** | **BF10** |
| --- | --- | --- | --- | --- | --- |
| **AC1** | *NAVON* | 0.49 | 0.20 | 0.68 | 30.5 |
|  | *Posner* | 0.52 | 0.23 | 0.70 | 52.6 |
|  | *SNARC* | 0.36 | 0.04 | 0.59 | 2.24 |
|  | *Flanker* | 0.25 | 0.06 | 0.42 | 2.85 |
|  | *Go/Nogo* | 0.27 | 0.08 | 0.43 | 5.35 |
|  | *Stroop* | 0.19 | -0.01 | 0.37 | 0.75 |
|  | *Stop-signal* | 0.23 | 0.04 | 0.41 | 1.77 |
| **PSD** | *NAVON* | 0.23 | -0.08 | 0.49 | 0.55 |
|  | *Posner* | 0.45 | 0.15 | 0.65 | 11.77 |
|  | *SNARC* | 0.28 | -0.03 | 0.53 | 0.87 |
|  | *Flanker* | 0.23 | 0.04 | 0.40 | 1.89 |
|  | *Go/Nogo* | 0.31 | 0.12 | 0.47 | 16.87 |
|  | *Stroop* | 0.23 | 0.04 | 0.40 | 1.73 |
|  | *Stop-signal* | 0.34 | 0.15 | 0.50 | 49.22 |
| **DFA** | *NAVON* | 0.37 | 0.06 | 0.60 | 3.00 |
|  | *Posner* | 0.1 | -0.21 | 0.38 | 0.23 |
|  | *SNARC* | 0.21 | -0.1 | 0.48 | 0.45 |
|  | *Flanker* | 0.16 | -0.03 | 0.34 | 0.47 |
|  | *Go/Nogo* | 0.06 | -0.13 | 0.25 | 0.15 |
|  | *Stroop* | 0.22 | 0.02 | 0.39 | 1.32 |
|  | *Stop-signal* | 0.31 | 0.12 | 0.48 | 16.70 |
| **d** | *NAVON* | 0.003 | -0.30 | 0.30 | 0.20 |
|  | *Posner* | 0.26 | -0.05 | 0.52 | 0.72 |
|  | *SNARC* | 0.05 | -0.26 | 0.34 | 0.21 |
|  | *Flanker* | 0.16 | -0.04 | 0.34 | 0.43 |
|  | *Go/Nogo* | 0.05 | -0.14 | 0.24 | 0.14 |
|  | *Stroop* | -0.07 | -0.26 | 0.12 | 0.16 |
|  | *Stop-signal* | 0.16 | -0.04 | 0.34 | 0.44 |
| **AR** | *NAVON* | 0.07 | -0.24 | 0.36 | 0.22 |
|  | *Posner* | 0.18 | -0.14 | 0.45 | 0.36 |
|  | *SNARC* | -0.25 | -0.5 | 0.07 | 0.61 |
|  | *Flanker* | 0.07 | -0.13 | 0.25 | 0.15 |
|  | *Go/Nogo* | 0.01 | -0.18 | 0.26 | 0.12 |
|  | *Stroop* | 0.01 | -0.18 | 0.2 | 0.13 |
|  | *Stop-signal* | 0.17 | -0.03 | 0.35 | 0.49 |
| **MA** | *NAVON* | 0.02 | -0.28 | 0.32 | 0.2 |
|  | *Posner* | 0.21 | -0.11 | 0.47 | 0.43 |
|  | *SNARC* | -0.23 | -0.49 | 0.08 | 0.54 |
|  | *Flanker* | 0.13 | -0.06 | 0.31 | 0.29 |
|  | *Go/Nogo* | -0.001 | -0.12 | 0.26 | 0.12 |
|  | *Stroop* | 0.0003 | -0.19 | 0.19 | 0.12 |
|  | *Stop-signal* | 0.13 | -0.07 | 0.31 | 0.27 |

*Table A2. Full list of the correlation coefficients (r-value) with corresponding 95% CI ([lower, upper]) and Bayes Factor (BF10) for the between-task repeatability of cognitive tasks, as displayed in Figure 6.*

| **Measure** | **Task 1** | **Task 2** | **r-value** | **lower** | **upper** | **BF10** |
| --- | --- | --- | --- | --- | --- | --- |
| **AC1** | *NAVON* | *Posner* | 0.11 | -0.21 | 0.39 | 0.24 |
|  |  | *SNARC* | 0.39 | 0.09 | 0.61 | 4.14 |
|  | *Posner* | *SNARC* | 0.29 | -0.03 | 0.54 | 0.94 |
|  | *Flanker* | *Go/nogo* | 0.17 | -0.07 | 0.31 | 0.27 |
|  |  | *Stroop* | 0.001 | -0.19 | 0.19 | 0.12 |
|  |  | *Stop-signal* | 0.03 | -0.16 | 0.23 | 0.13 |
|  | *Go/Nogo* | *Stroop* | 0.25 | 0.06 | 0.42 | 3.23 |
|  |  | *Stop-signal* | -0.05 | -0.24 | 0.15 | 0.14 |
|  | *Stroop* | *Stop-signal* | 0.14 | -0.06 | 0.32 | 0.30 |
|  | *SART* | *Visual Search* | 0.12 | -0.24 | 0.44 | 0.28 |
| **PSD** | *NAVON* | *Posner* | 0.34 | 0.03 | 0.58 | 1.89 |
|  |  | *SNARC* | 0.32 | 0.002 | 0.56 | 1.31 |
|  | *Posner* | *SNARC* | 0.30 | -0.01 | 0.55 | 1.11 |
|  | *Flanker* | *Go/nogo* | 0.12 | -0.07 | 0.30 | 0.26 |
|  |  | *Stroop* | 0.08 | -0.12 | 0.26 | 0.17 |
|  |  | *Stop-signal* | 0.02 | -0.18 | 0.21 | 0.13 |
|  | *Go/Nogo* | *Stroop* | 0.27 | 0.08 | 0.44 | 5.40 |
|  |  | *Stop-signal* | 0.12 | -0.08 | 0.31 | 0.26 |
|  | *Stroop* | *Stop-signal* | 0.07 | -0.13 | 0.26 | 0.16 |
|  | *SART* | *Visual Search* | 0.02 | -0.33 | 0.36 | 0.23 |
| **DFA** | *NAVON* | *Posner* | 0.13 | -0.19 | 0.41 | 0.27 |
|  |  | *SNARC* | 0.07 | -0.24 | 0.36 | 0.21 |
|  | *Posner* | *SNARC* | 0.19 | -0.12 | 0.46 | 0.39 |
|  | *Flanker* | *Go/nogo* | -0.03 | -0.22 | 0.16 | 0.13 |
|  |  | *Stroop* | 0.20 | 0.003 | 0.37 | 0.87 |
|  |  | *Stop-signal* | 0.02 | -0.17 | 0.22 | 0.13 |
|  | *Go/Nogo* | *Stroop* | 0.11 | -0.08 | 0.29 | 0.23 |
|  |  | *Stop-signal* | 0.04 | -0.16 | 0.23 | 0.14 |
|  | *Stroop* | *Stop-signal* | 0.10 | -0.10 | 0.29 | 0.20 |
|  | *SART* | *Visual Search* | 0.12 | -0.24 | 0.44 | 0.28 |
| **d** | *NAVON* | *Posner* | -0.12 | -0.41 | 0.19 | 0.26 |
|  |  | *SNARC* | 0.17 | -0.15 | 0.44 | 0.33 |
|  | *Posner* | *SNARC* | -0.20 | -0.47 | 0.12 | 0.40 |
|  | *Flanker* | *Go/nogo* | 0.07 | -0.13 | 0.25 | 0.16 |
|  |  | *Stroop* | 0.25 | 0.06 | 0.42 | 3.22 |
|  |  | *Stop-signal* | -0.04 | -0.23 | 0.16 | 0.14 |
|  | *Go/Nogo* | *Stroop* | 0.08 | -0.12 | 0.26 | 0.17 |
|  |  | *Stop-signal* | -0.01 | -0.21 | 0.18 | 0.13 |
|  | *Stroop* | *Stop-signal* | 0.06 | -0.14 | 0.26 | 0.15 |
|  | *SART* | *Visual Search* | 0.08 | -0.27 | 0.41 | 0.25 |
| **AR** | *NAVON* | *Posner* | -0.11 | -0.40 | 0.20 | 0.25 |
|  |  | *SNARC* | 0.46 | 0.16 | 0.66 | 14.3 |
|  | *Posner* | *SNARC* | -0.18 | -0.46 | 0.13 | 0.36 |
|  | *Flanker* | *Go/nogo* | 0.01 | -0.18 | 0.20 | 0.12 |
|  |  | *Stroop* | 0.11 | -0.09 | 0.30 | 0.22 |
|  |  | *Stop-signal* | -0.04 | -0.23 | 0.16 | 0.13 |
|  | *Go/Nogo* | *Stroop* | 0.07 | -0.12 | 0.26 | 0.16 |
|  |  | *Stop-signal* | 0.05 | -0.15 | 0.24 | 0.14 |
|  | *Stroop* | *Stop-signal* | 0.07 | -0.13 | 0.26 | 0.16 |
|  | *SART* | *Visual Search* | -0.06 | -0.39 | 0.29 | 0.24 |
| **MA** | *NAVON* | *Posner* | -0.04 | -0.34 | 0.26 | 0.20 |
|  |  | *SNARC* | 0.42 | 0.12 | 0.63 | 6.82 |
|  | *Posner* | *SNARC* | -0.31 | -0.55 | 0.01 | 1.16 |
|  | *Flanker* | *Go/nogo* | 0.02 | -0.17 | 0.21 | 0.13 |
|  |  | *Stroop* | 0.12 | -0.08 | 0.30 | 0.24 |
|  |  | *Stop-signal* | -0.01 | -0.20 | 0.19 | 0.13 |
|  | *Go/Nogo* | *Stroop* | 0.07 | -0.12 | 0.26 | 0.16 |
|  |  | *Stop-signal* | 0.0003 | -0.20 | 0.19 | 0.13 |
|  | *Stroop* | *Stop-signal* | 0.07 | -0.13 | 0.26 | 0.16 |
|  | *SART* | *Visual Search* | -0.1 | -0.42 | 0.26 | 0.26 |

**Supplementary Materials B**

*Table B. Pearson r-values between the different measures of temporal dependency on each of the archival datasets, with each row reflecting one correlation pair.* *Green and red fonts indicate clear evidence against and for the null respectively; black font indicates no reliable conclusion can be drawn.*

|  | **MRT** | **SART** | **VS** | **Flanker** | **Stroop** | **Stopsignal** | **Go/No-go** | **NAVON** | **SNARC** | **Posner** |
| --- | --- | --- | --- | --- | --- | --- | --- | --- | --- | --- |
| **AC1-PSD** | .93 | .92 | .87 | .89 | .88 | .76 | .89 | .77 | .91 | .85 |
| **AC1-DFA** | .77 | .66 | .55 | .60 | .69 | .68 | .67 | .86 | .57 | .59 |
| **AC1-d** | .04 | .23 | .16 | .31 | .37 | .50 | .33 | -.26 | .16 | .41 |
| **AC1-AR** | .26 | -.09 | .07 | -.02 | -.10 | .13 | .18 | .20 | -.01 | -.18 |
| **AC1-MA** | .07 | -.17 | -.07 | -.12 | -.12 | .13 | .12 | .04 | -.07 | -.19 |
| **PSD-DFA** | .69 | .62 | .59 | .57 | .68 | .72 | .64 | .64 | .56 | .65 |
| **PSD-d** | -.02 | .22 | .09 | .22 | .23 | .31 | .28 | -.41 | .03 | .32 |
| **PSD-AR** | .22 | -.17 | .10 | .03 | .19 | .02 | .14 | .18 | -.07 | -.18 |
| **PSD-MA** | -.01 | -.26 | -.02 | -.06 | .22 | .04 | .07 | -.03 | -.15 | -.20 |
| **DFA-d** | .10 | .32 | .16 | .48 | .33 | .59 | .46 | -.19 | .51 | .57 |
| **DFA-AR** | .19 | -.04 | -.07 | -.16 | -.15 | -.04 | .18 | -.02 | -.25 | -.12 |
| **DFA-MA** | .12 | -.04 | -.11 | -.15 | -.13 | .04 | .20 | -.14 | -.18 | -.04 |
| **d-AR** | -.59 | .03 | -.44 | -.55 | -.18 | .21 | -.25 | -.41 | -.07 | -.02 |
| **d-MA** | -.27 | .22 | -.27 | -.39 | .03 | .41 | -.08 | -.12 | .13 | .22 |
| **ar-MA** | .94 | .97 | .97 | .96 | .97 | .97 | .98 | .95 | .97 | .96 |

**Supplementary Materials C**

*Table C1. Within- and between-task repeatability analyses using different analysis choices, in which the missing values in the RT series were replaced either by the median value or highest possible (MRT only) value, or in which the frequency was capped (PSD and DFA only). Shown are the r-values of the correlation analyses, with green and red fonts indicating clear evidence against and for the null respectively. R-values which Bayesian evidence has changed evidence are highlighted in dark grey, and r-values which evidence did not change but were at least .10 higher or lower than the original estimate are highlighted in light grey.*

|  | **AC1** | **PSD** | **DFA** | **AR** | **MA** | **d** |
| --- | --- | --- | --- | --- | --- | --- |
| **Within-task: median replacement** | | |  |  |  |  |
| MRT both cohorts | .82 | .78 | .56 | .09 | .08 | .32 |
| Flanker | .25 | .23 | .16 | .07 | .13 | 16 |
| Go/No-go | .31 | .27 | .25 | .08 | .01 | .18 |
| Stroop | .19 | .23 | .22 | .01 | -.01 | -.07 |
| Stop-signal | .23 | .34 | .31 | .17 | .13 | .16 |
| NAVON | .49 | .23 | .37 | .07 | .02 | .003 |
| Posner | .50 | .37 | .04 | .30 | .29 | .29 |
| SNARC | .36 | .28 | .21 | .05 | -.25 | -.23 |
|  |  |  |  |  |  |  |
| **Within-task: capped** | | |  |  |  |  |
| MRT both cohorts |  | .58 | .44 |  |  |  |
| Flanker |  | .23 | .10 |  |  |  |
| Go/No-go |  | .28 | .01 |  |  |  |
| Stroop |  | .23 | .15 |  |  |  |
| Stop-signal |  | .27 | .32 |  |  |  |
| NAVON |  | .19 | .38 |  |  |  |
| Posner |  | .40 | -.22 |  |  |  |
| SNARC |  | .32 | .21 |  |  |  |
|  |  |  |  |  |  |  |
| **Within-task: high replacement** | |  |  |  |  |  |
| MRT both cohorts | .53 | .53 | .38 | .24 | .05 | .33 |
|  |  |  |  |  |  |  |
|  |  |  |  |  |  |  |
|  |  |  |  |  |  |  |
| **Between-task: median replacement** | | |  |  |  |  |
| SART-VS | .11 | .16 | .03 | -.39 | -.37 | .27 |
| NAVON-Posner | .10 | .23 | .09 | -.14 | -.08 | -.14 |
| NAVON-SNARC | .39 | .32 | .07 | .46 | .42 | .03 |
| Posner-SNARC | .26 | .26 | .21 | -.19 | -.33 | -.18 |
| Flanker-Go/Nogo | .12 | .12 | .10 | .06 | .05 | .08 |
| Flanker-Stroop | .001 | .08 | .20 | .11 | .12 | .25 |
| Flanker-Stop | .03 | .02 | .02 | -.04 | -.01 | -.04 |
| Go/Nogo-Stroop | .28 | .23 | .13 | -.08 | -.03 | .01 |
| Go/Nogo-Stop | .09 | .04 | .05 | -.02 | -.06 | -.10 |
| Stroop-Go/Stop | .14 | .07 | .10 | .07 | .07 | .06 |
|  |  |  |  |  |  |  |
| **Between-task: capped** |  |  |  |  |  |  |
| SART-VS |  | .02 | .12 |  |  |  |
| NAVON-Posner |  | .29 | -.10 |  |  |  |
| NAVON-SNARC |  | .35 | .06 |  |  |  |
| Posner-SNARC |  | .41 | .03 |  |  |  |
| Flanker-Go/Nogo |  | .10 | -.03 |  |  |  |
| Flanker-Stroop |  | .06 | .11 |  |  |  |
| Flanker-Stop |  | -.05 | .02 |  |  |  |
| Go/Nogo-Stroop |  | .24 | .07 |  |  |  |
| Go/Nogo-Stop |  | .13 | .07 |  |  |  |
| Stroop-Go/Stop |  | .08 | .13 |  |  |  |

*Table C2. Between-subject correlation analyses between temporal dependency and task measures from the MRT using different analysis choices. Conventions are the same as in Table B2.*

|  | **AC1** | **PSD** | **DFA** | **AR** | **MA** | **d** |
| --- | --- | --- | --- | --- | --- | --- |
| **Performance: median replacement** | | |  |  |  |  |
| MRT both cohorts | .58 | .58 | .39 | .13 | .03 | .12 |
| Archival MRT | .07 | .13 | -.23 | -.11 | -.18 | .01 |
|  |  |  |  |  |  |  |
| **Performance: capped** | | |  |  |  |  |
| MRT both cohorts |  | .44 | .10 |  |  |  |
| Archival MRT |  | -.13 | -.41 |  |  |  |
|  |  |  |  |  |  |  |
| **Performance: high replacement** | |  |  |  |  |  |
| MRT both cohorts | .45 | .48 | .40 | .26 | .23 | .19 |
| Archival MRT | -.11 | .09 | -.31 | .28 | .23 | -.37 |
|  |  |  |  |  |  |  |
|  |  |  |  |  |  |  |
| **Mean attentional state: median replacement** | | | |  |  |  |
| MRT both cohorts | -.06 | -.08 | -.05 | -.02 | -.01 | .002 |
| Archival MRT | -.26 | -.22 | -.20 | -.09 | -.04 | .03 |
|  |  |  |  |  |  |  |
| **Mean attentional state: capped** | | |  |  |  |  |
| MRT both cohorts |  | .002 | -.07 |  |  |  |
| Archival MRT |  | -.21 | -.08 |  |  |  |
|  |  |  |  |  |  |  |
| **Mean attentional state: high replacement** | | |  |  |  |  |
| MRT both cohorts | .01 | .01 | .04 | .05 | .05 | .004 |
| Archival MRT | -.28 | -.25 | -.24 | -.02 | .07 | -.08 |
|  |  |  |  |  |  |  |
|  |  |  |  |  |  |  |
| **SD attentional state: median replacement** | | | |  |  |  |
| MRT both cohorts | .05 | .07 | .02 | .10 | .10 | .08 |
| Archival MRT | -.10 | -.10 | -.07 | -.01 | .01 | -.06 |
|  |  |  |  |  |  |  |
| **SD attentional state: capped** | | |  |  |  |  |
| MRT both cohorts |  | .16 | -.10 |  |  |  |
| Archival MRT |  | -.09 | -.05 |  |  |  |
|  |  |  |  |  |  |  |
| **SD attentional state: high replacement** | | |  |  |  |  |
| MRT both cohorts | .07 | .08 | .06 | .03 | .03 | -.01 |
| Archival MRT | -.15 | -.14 | -.13 | .003 | .03 | -.10 |

*Table C3. Between-subject correlation analyses between temporal dependency and questionnaire scores using different analysis choices. Conventions are the same as above.*

|  | **AC1** | **PSD** | **DFA** |
| --- | --- | --- | --- |
| **Median replacement** | | |  |
| ADHD | .04 | .01 | .06 |
| DFS | -.03 | -.08 | .06 |
| UPPS-P | -.02 | -.06 | -.02 |
| ARCES | -.03 | -.01 | -.03 |
|  |  |  |  |
| **Capped** |  |  |  |
| ADHD |  | -.04 | -.06 |
| DFS |  | -.04 | -.06 |
| UPPS-P |  | -.09 | .06 |
| ARCES |  | .002 | -.09 |
|  |  |  |  |
| **High replacement** |  |  |  |
| ADHD | -.04 | -.03 | .02 |
| DFS | .01 | -.05 | -.02 |
| UPPS-P | -.07 | -.11 | -.09 |
| ARCES | -.07 | -.07 | -.09 |
